# Peptide isolated from Cry1Ab16 toxin present in *Bacillus thuringiensis*: Synthesis and morphology data for layer-by-layer films studied by atomic force microscopy

**DOI:** 10.1016/j.dib.2016.05.031

**Published:** 2016-05-21

**Authors:** Alexandra Plácido, Emanuel Airton de Oliveira Farias, Mariela M. Marani, Andreanne Gomes Vasconcelos, José R.S.A. Leite, Cristina Delerue-Matos

**Affiliations:** a REQUIMTE/LAQV, Instituto Superior de Engenharia do Porto, ISEP, Instituto Politécnico do Porto, Porto, Portugal; b Núcleo de Pesquisa em Biodiversidade e Biotecnologia, BIOTEC, Campus Ministro Reis Velloso, CMRV, Universidade Federal do Piauí, UFPI, Parnaíba, PI, Brasil; c IPEEC-CONICET, Consejo Nacional de Investigaciones Científicas y Técnicas, Puerto Madryn, Chubut, Argentina

**Keywords:** Cry1Ab16 toxin, *Bacillus thuringiensis*, Layer-by-layer films, Atomic force microscopy, GMO׳s

## Abstract

The peptide PcL342-354C was obtained from the Cry1Ab16 toxin present in *Bacillus thuringiensis* (“Computational Modeling Deduced Three Dimensional Structure of Cry1Ab16 Toxin from *B. thuringiensis* AC11” (Kashyap, 2012) [1]). In this data article, we report the synthesis and characterization of the PcL342-354C peptide by MALDI-TOF/TOF mass spectrometry. In addition, the preparation of layer-by-layer films is shown based on interspersion of this peptide with both polyethylenimine (PEI) and poly(sodium 4-styrenesulfonate) (PSS), self-assembled on ITO (indium tin oxide) electrodes. The morphology of the ITO/PEI/PSS/PcL342-354C film was analyzed using atomic force microscopy (AFM). We also evaluated the effect of the number of bilayers in ITO/PEI/(PSS/PcL342-354C)*_n_* on the morphology of the film using AFM amplitude images. Further details about this study were published elsewhere, “Layer-by-layer films containing peptides of the Cry1Ab16 toxin from *B. thuringiensis* for potential biotechnological applications,” (Plácido et al., 2016) [2].

**Specifications table**

**Table:** 

Subject area	*Chemistry, Food Technology, and Materials Science.*
More specific subject Area	*Biotechnology*
Type of data	*Figure*
How data was acquired	Fig. 1*(analytical RP-HPLC, Shimadzu Co.).*
	Fig. 2, Fig. 3*(MALDI-TOF/TOF mass spectrometry, UltrafleXtreme, Bruker Daltonics).*
	Fig. 4*(atomic force microscopy, TT-AFM, AFM Workshop; images were analyzed and displayed using Gwyddion 2.29 software).*
Data format	*Analyzed.*
Experimental factors	*Isolation of PcL342-354C peptide and preparation of LbL films containing this peptide.*
Experimental features	*Purification in the RP-HPLC system.*
	*Sequencing performed using LIFT mode in MALDI-TOF/TOF systems.*
	*Representative films examined using AFM. Analysis of the samples was carried out in vibrating (tapping) mode.*
Data source location	*Universidade Federal do Piauí, Parnaíba-PI, Brazil.*
Data accessibility	*Data is within this article.*

**Value of the data**

• The dataset provides details about the synthesis of a new peptide (PcL342-354C) produced from protein Cry1Ab16 [1], which is an insecticidal toxin that has been widely used in genetically modified organisms (GMOs). This peptide can be further used for development of antibodies against GMOs and enable the development of sensors for food safety.
• The data provides a method for producing self-assembled films (Layer-by-Layer) containing PcL342-354C and polymers used in the fabrication of sensors.
• For the first time, the morphology of LbL films based on PcL342-354C was observed by AFM. Morphology is an important aspect that should be considered during development of sensors.
• The films developed by our group have potential for future applications in GMOs sensors for applications in food safety (Fig. 1, Fig. 2, Fig. 3, Fig. 4).

## 1 Data

The data presented here provide details on the synthesis and purification of peptide PcL342-354C isolated from Cry1Ab16 insecticidal toxin. Additionally, characterization data of peptide by mass spectrometry is showed. The morphology of LbL films containing the peptide intercalated with PEI and PSS was evaluated by AFM. The produced films are potential candidates for use in GMO׳s sensors [2].

## 2 Experimental design, materials, and methods

### 2.1 Peptide synthesis and characterization

The PcL342-354C peptide were manually synthesized using a solid phase approach with Fmoc/t-butyl chemistry [3] and adapted by [4]. Peptide elongation was carried out in polypropylene syringes fitted with a polyethylene porous disk. Solvents and soluble reagents were removed by suction. A Cys residue was incorporated at the C-terminus in order to allow subsequent coupling. Samples were treated with trifluoroacetic acid/triisopropylsilane/water (TFA/TIS/H_2_O) (95:2.5:2.5) to remove the protecting group. Peptide purification was carried out using an analytical RP-HPLC system (Shimadzu Prominence, Shimadzu Co.) equipped with a reverse-phase (RP) chromatographic column (Kinetex 5 µm C18, 50×21.20 mm, Phenomenex). Peptides were monitored at 216 and 280 nm. The formula (*A*_215_−*A*_225_)×144 (µg/mL) was applied for peptide quantification [5]. Purity and molecular mass determination of synthetic peptides were performed using a MALDI-TOF/TOF mass spectrometer (UltrafleXtreme, Bruker Daltonics).

### 2.2 Layer-by-layer film preparation

The layer-by-layer (LbL) films prepared in this work were based on an electrostatic interactions between polyelectrolytes of different charges, as described by Decher (1997) [6]. A solution of polyethylenimine (PEI) was used as the polycation, and a solution of poly(sodium 4-styrenesulfonate) (PSS) was used as the polyanion. PEI was used to give the substrate a positive charge for better adsorption of PSS, whereas PSS was used to increase the adsorption of (positively charged) PcL342-354C. To obtain films, a previously cleaned ITO substrate [7] was immersed in the solution of PEI (1 mg/mL) for 5 min. Then, the substrate modified with a PEI monolayer was immersed in the solution of PSS, also for 5 min. Subsequently, the ITO/PEI/PSS film was immersed in the PcL342-354C solution for 5 min. After each adsorption step, the substrate was cleaned with ultrapure water and dried with nitrogen. The deposition time was tested and 5 min was found to allow a good electrochemical signal response. To obtain films with higher numbers of bilayers, the PSS and PcL342-354C adsorption processes were repeated until the desired number of bilayers was obtained.

### 2.3 Atomic force microscopy analysis

Representative films were examined using AFM. The analysis was carried on the samples in vibrating (tapping) mode. Imaging was performed using a TT-AFM instrument (AFM Workshop). All images were collected at 512 pixel resolution for a 4×4 µm area, and at least three different areas were examined per sample. The images have been processed, analyzed, and displayed using Gwyddion 2.29 software [8].

## Figures and Tables

**Fig. 1:**
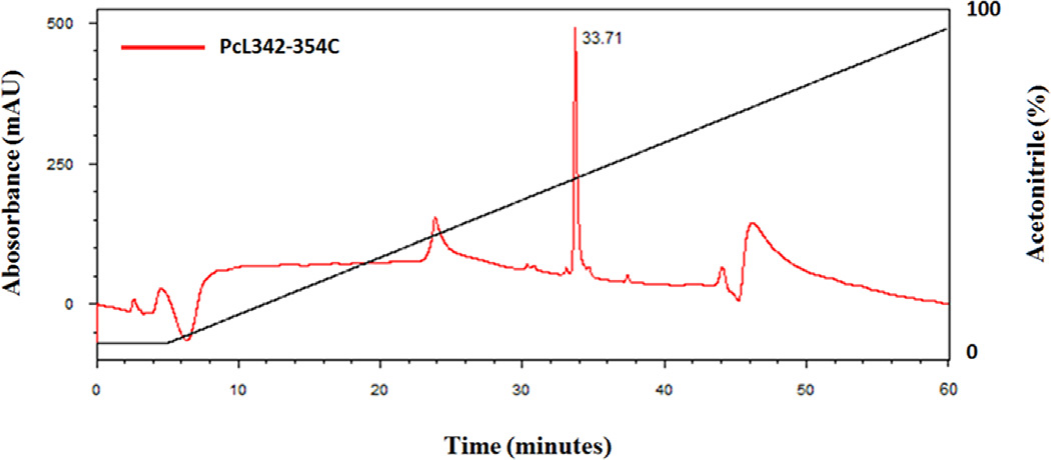
Purification of the PcL342-354C peptide in the analytical RP-HPLC system.

**Fig. 2:**
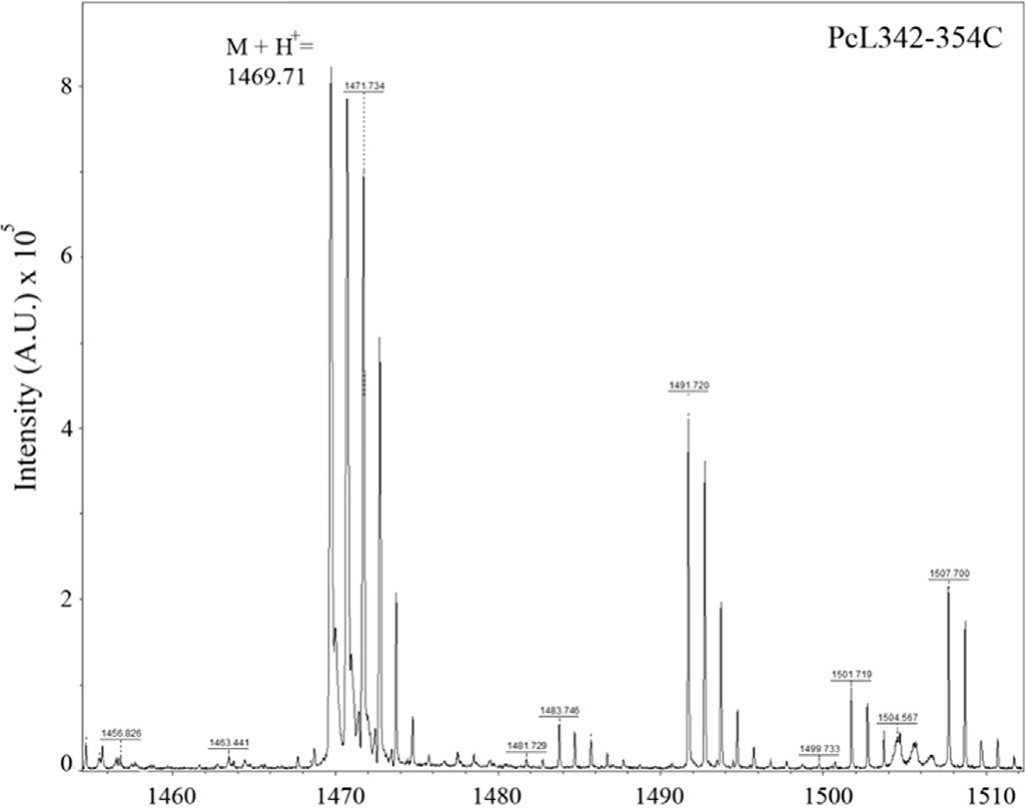
MS analysis of the PcL342-354C peptide ([M+H]^+^=1469.71 Da).

**Fig. 3:**
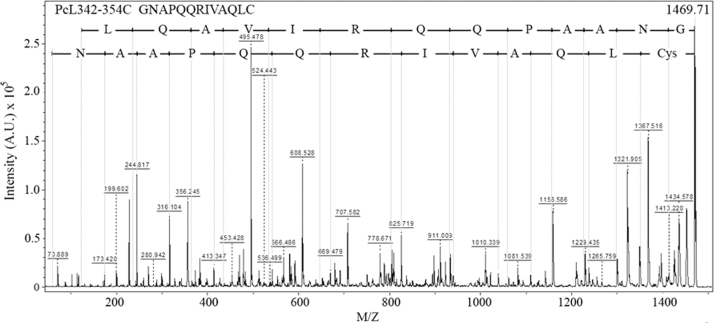
Analysis of the MS/MS spectrum of the PcL342-354C peptide.

**Fig. 4:**
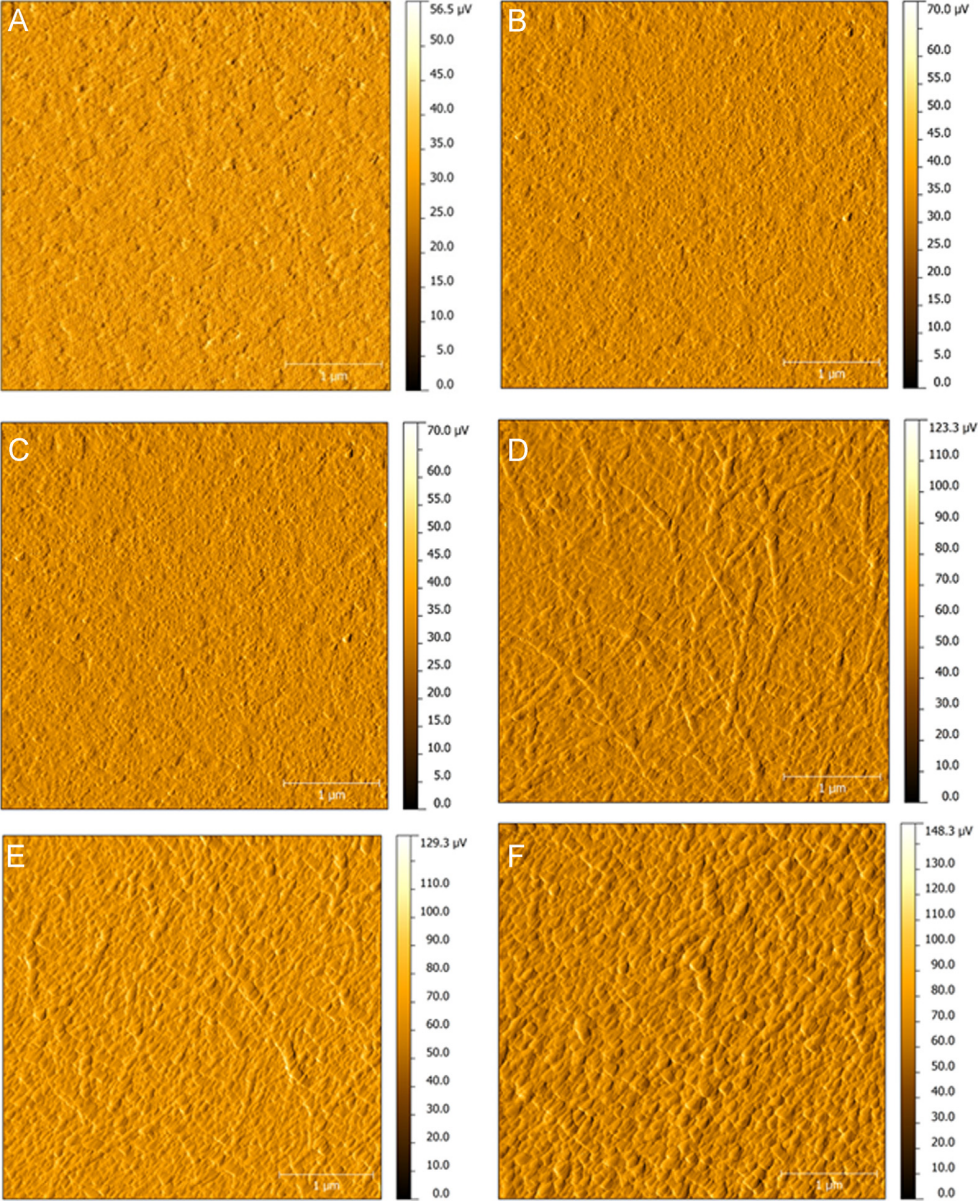
AFM amplitude images for glass surfaces covered with: (A) ITO, LbL films of (B) ITO/PEI, (C) ITO/PEI/PSS, (D) ITO/PEI/PSS/PcL342-354C, (E) ITO/PEI/(PSS/PcL342-354C)_2_, and (F) ITO/PEI/(PSS/PcL342-354C)_5_. All images are 4×4 µm in *x* and *y*.

## References

[1] Kashyap S. (2012). Computational modeling deduced three dimensional structure of Cry1Ab16 toxin from *bacillus thuringiensis* AC11. Indian J. Microbiol..

[2] Plácido A., Farias E.A.O., Marani M.M., Vasconcelos A.G., Mafud A.C., Mascarenhas Y.P., Eiras C., Leite J.R.S.A., Delerue-Matos C. (2016). Layer-by-layer films containing peptides of the Cry1Ab16 toxin from *Bacillus thuringiensis* for potential biotechnological applications. Mater. Sci. Eng.: C.

[3] Merrifield R.B. (1963). Solid phase peptide synthesis. I. The synthesis of a tetrapeptide. J. Am. Chem. Soc..

[4] Marani M.M., Dourado Fv.S., Quelemes P.V., de Araujo A.R., Perfeito Mr.L.G., Barbosa E.A., Véras L.M.C., Coelho A.L.R., Andrade E.B., Eaton P. (2015). Characterization and biological activities of ocellatin peptides from the skin secretion of the frog Leptodactylus pustulatus. J. Nat. Prod..

[5] Wolf P. (1983). A critical reappraisal of Waddell׳s technique for ultraviolet spectrophotometric protein estimation. Anal. Biochem..

[6] Decher G. (1997). Fuzzy nanoassemblies: toward layered polymeric multicomposites. science.

[7] Kern W. (1984). Purifying Si and SiO_2_ surfaces with hydrogen peroxide. Semicond. Int..

[8] Eaton P., West P. (2010). Atomic Force Microscopy.

